# A Fully Immersive Set-Up for Remote Interaction and Neurorehabilitation Based on Virtual Body Ownership

**DOI:** 10.3389/fneur.2012.00110

**Published:** 2012-07-10

**Authors:** Daniel Perez-Marcos, Massimiliano Solazzi, William Steptoe, Oyewole Oyekoya, Antonio Frisoli, Tim Weyrich, Anthony Steed, Franco Tecchia, Mel Slater, Maria V. Sanchez-Vives

**Affiliations:** ^1^Institut d’Investigacions Biomèdiques August Pi i SunyerBarcelona, Spain; ^2^Experimental Virtual Environments for Neuroscience and Technology-Lab, Facultat de Psicologia, Universitat de BarcelonaBarcelona, Spain; ^3^Perceptual Robotics Laboratory, Scuola Superiore Sant’AnnaPisa, Italy; ^4^Department of Computer Science, University College LondonLondon, UK; ^5^Institució Catalana Recerca i Estudis AvançatsBarcelona, Spain

**Keywords:** virtual reality, telemedicine, teleneurology, neurorehabilitation, body ownership, multisensory correlations, haptics, rubber hand illusion

## Abstract

Although telerehabilitation systems represent one of the most technologically appealing clinical solutions for the immediate future, they still present limitations that prevent their standardization. Here we propose an integrated approach that includes three key and novel factors: (a) fully immersive virtual environments, including virtual body representation and ownership; (b) multimodal interaction with remote people and virtual objects including haptic interaction; and (c) a physical representation of the patient at the hospital through embodiment agents (e.g., as a physical robot). The importance of secure and rapid communication between the nodes is also stressed and an example implemented solution is described. Finally, we discuss the proposed approach with reference to the existing literature and systems.

## Introduction

Neurorehabilitation facilitates the recovery of functional skills lost after neurological diseases or accidents. According to the American Academy of Neurology, “neurorehabilitation is the process of restoration of function for persons with disorders of the nervous system. This process involves multiple disciplines and the application of strategies aimed at reducing impairments, disabilities and handicaps, and ultimately enhancing quality of life for persons with neurological disorders.” Neurorehabilitation is an emerging specialty in Neurology (Dimyan et al., 2008), and the integration of technology at this frontier is of interest from both a medical (Levin, 2011) and social perspective, and is particularly relevant when the rehabilitation has to be performed at home due to limitations of patient mobility (Cranen et al., 2011). Therefore, it represents a field with high expectations for the future, where the integration of new technologies may enhance the versatility and effectiveness of the current rehabilitation systems (Levin, 2011).

### Telepresence and neurorehabilitation

In recent years, neurorehabilitation has found in telepresence (Minsky, 1980; Steuer, 1992) a convenient and promising ally. Telerehabilitation systems allow remote assistance, which may reduce the stress of a visit to the hospital (Cranen et al., 2011) or the pain in patients with acute or chronic pain (Golomb et al., 2010). Patients with reduced mobility can benefit from the possibility of remote interaction with their doctors and other patients, and also carry out this training from their home (Golomb et al., 2010), under remote supervision. For doctors, telerehabilitation systems provide online remote monitoring of both the rehabilitation process, the clinical and physiological parameters of the patient, and the personal interaction in a virtual space (Holden et al., 2007; Leon et al., 2011).

### Virtual reality and neurorehabilitation

Virtual reality (VR) can provide the appropriate experience to support remote rehabilitation (Burdea, 2003; Levin, 2011; Saposnik and Levin, 2011). By VR we refer to a set of technologies that attempts to create an immersive computer display that surrounds the participant (Ellis, 1991). VR replaces direct vision and audition of the real environment with synthesized stimuli, and can also integrate haptic (tactile and force) cues representing virtual objects or remote interactions (Popescu et al., 2000; August et al., 2011). In remote teleneurology, the exploitation of VR is able to provide real time feedback to the participant (Merians et al., 2002; Cameirao et al., 2009), comprised of parallel streams of sensory information (visual, sound, or haptics; Adamovich et al., 2009b). The current proliferation of VR-based telerehabilitation systems is reviewed in (Brochard et al., 2010), and has enabled new paths for the development of multimodal scenarios supporting multisensory interaction in both independent and collaborative scenarios.

The capacity of VR-based systems as a facilitation tool for functional recovery by engaging brain circuits, such as motor areas, has been demonstrated (Adamovich et al., 2009b). A recent review study has shown that such systems can be effective and motivating for rehabilitation therapies involving repetition and feedback (Holden, 2005). It seems that motivation is a key factor for applications based on augmented feedback using VR for rehabilitation of motor skills of patients with neurological disorders (Robertson and Roby-Brami, 2010). In particular, there is evidence for the effectiveness of such approaches for the rehabilitation of upper limbs in patients with stroke (Crosbie et al., 2007; Henderson et al., 2007; Lucca, 2009; Saposnik and Levin, 2011).

### The importance of virtual body ownership

Apart from immersion and motivation, critical ability of VR in the context of neurorehabilitation is the possibility to induce ownership of a virtual body. The term “body ownership” encompasses a number of illusions that rely on the remarkable plasticity of the brain to accept altered representation or even other objects as part of our own body (Ehrsson et al., 2004). A body illusion of ownership arises when someone feels an external, fake, or virtual body part to be part of their own body. The generation of such an illusion is based on providing synchronous multisensory or sensorimotor correlations coherent with the ownership of the fake body part. This requires at least more than one sensory modality: visual perception, tactile, proprioceptive, vestibular, or kinesthetic. The classical example is the simple and static rubber hand that replaces (and is felt as) the own hand. This occurs when both the real and fake hands are synchronously touched (in spatially equivalent locations and synchronously in time), and the person only sees the touch on the rubber hand aligned with the real (hidden) one (Botvinick and Cohen, 1998). This evidence has been extended to 3D computer-generated virtual body parts such as virtual hands (Slater et al., 2008, 2009) or virtual belly (Normand et al., 2011). The fact that a virtual body part can be incorporated into the body schema based on synchronous visuo-tactile correlations has opened new paths for examining the mechanisms of body perception. Body illusions can be induced not only with specific body parts but also with the body as a whole as in (Ehrsson, 2007; Lenggenhager et al., 2007; Slater et al., 2010). In those experiments, synchronous visuo-tactile stimulation on the upper body of the participant and a video or virtual representation of the participants represented in front of themselves by means of a head-mounted display (HMD) resulted in a proprioceptive displacement toward the virtual representation. Participants felt the touches on the upper body as if they came from the same location from their visual representation rather than their real body.

Multisensory integration based on visuo-tactile correlations is the most commonly used combination but not the only one. Indeed it has been suggested that only the visual input of a virtual body co-located with the real one while seen from a first-person point of view is enough to generate that feeling of virtual body ownership to a great extent (Slater et al., 2010), as long as the multisensory contingencies do not break. The strength of the illusion is reinforced when, to the visual co-location, synchronous visuo-motor correlations are provided, e.g., with the person controlling the body movements (arms, legs, etc.) of the avatar, who mimics her movements (Gonzalez-Franco et al., 2010; Sanchez-Vives et al., 2010).

Feeling a virtual body to be your own allows body transformation and manipulation in a way that it is not possible to do outside of VR (Normand et al., 2011). It has also been shown that illusory body experiences are able to induce similar levels of activity in the brain areas associated with anxiety and interoceptive awareness, as when the person’s real hand is threatened (Ehrsson et al., 2007), or lateralized autonomic responses such as changes in body temperature (Moseley et al., 2008).

In clinical terms, manipulations of a virtual body could have implications not only for motor or sensory rehabilitation but also for psychological treatment in different pathologies involving body perception, such as painful phantom limbs, regional pain syndrome (Llobera et al., submitted), eating disorders (Perpiñá et al., 1999; Riva, 2008), or burns (Hoffman et al., 2000). Likewise, current rehabilitation strategies may take advantage and enhance their performance ratios due to the holistic (mental and physical) engagement of patients who, unlike while using non-immersive systems, become main actors where the local or remote events are related to them, because they feel that what it is happening is real. This factor has been recently identified and defined as “plausibility” (Slater, 2009). Inducing ownership of a virtual body will also allow spatial collaboration with persons that are remotely located, e.g., in the case that we present here, medical personnel.

### Haptics, robotics, and neurorehabilitation

Neurorehabilitation borrows elements from the fields of haptics and robotics. Therapies based on telerehabilitation mainly employ haptic devices for monitoring data captured during physical exercises, so that the performance of patients can be evaluated (Adamovich et al., 2009a). However, haptic feedback also enriches sensory experience for the participant (August et al., 2011) by providing forces that produce biomechanical interactions with other devices, virtual objects (simulating the interaction forces produced by the same object in reality) or remotely located people (Popescu et al., 2000). These telerehabilitation set-ups can yield comparable benefits compared to those of traditional non-mediated therapies. For example, it has been shown that the effect of robot-mediated therapy can have greater effect than the same duration of non-functional exercises (Coote et al., 2008). A different range of robotic integration in rehabilitation are exoskeletons, which adapt their force to the patient’s performance and complement it to reach the defined goal, e.g., for gaiting purposes. For instance, Wolbrecht et al. (2008) have demonstrated in an experiment with chronic stroke patients that an adaptive “assist-as-needed” robot controller increases participation from the motor system. However, first clinical trials suggest that the only contribution to clinical practice currently is the provision of intensive, repetitive movements (Brochard et al., 2010; Lo et al., 2010). Moreover, a recent comparative study has shown that the advantages in both functioning recovery among patients with chronic upper-extremity disability related to stroke and cost-effectiveness of robot-assisted therapies are modest (Wagner et al., 2011).

Taking all these experiences together, there seems to be a general agreement that the field of robotic-assisted and telerehabilitation has yet not matured, as reflected by the considerable list of review papers published during the last years (see citations above) that discuss the future of VR, robotics, or remote rehabilitation. Therefore, despite the many benefits that these technologies potentially may provide in the clinic, this appealing technological approach has not so far yielded the expected improvement for rehabilitation therapies (Carignan and Krebs, 2006). In our opinion, beside the limitations of the current technology (in particular the still emerging field of haptics), there are several challenges that have been neglected up to now.

For successful telerehabilitation we argue that the environment should be highly physically and psychologically involving, meaning that the virtual experience is perceived as reality, i.e., ideally providing all sensory streams. The participant should become main actor in this reality rather than remaining as an external (although active) spectator as it is the case in video-games.

This paper describes an integrated system for telerehabilitation, which results of the integration of state of the art systems developed within the frame of an interdisciplinary and multicenter research project. The key elements that make this system unique among its peers are: (a) full immersion (mental and physical) into the virtual environment, by means of representation of the patient with a virtual body that is felt as their own; (b) multimodal (auditory, visual, tactile) interaction with remote people (doctors, patients, nurses); and (c) physical presence of the participant at the remote place through embodiment agents (e.g., as a physical robot). The aim of such a setting is then to produce a new kind of experience, where the person can be physically embodied interacting with people remotely. The result of that integration work is a scenario with relevance in clinical and rehabilitation environments. We next present a set-up for neurorehabilitation that integrates these elements.

## Description of a Set-Up for Remote Neurorehabilitation

**Figure 1:**
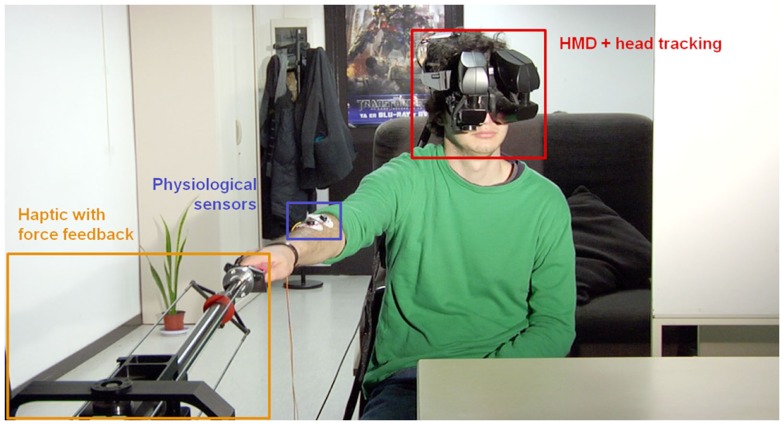
**Set-up at the patient’s home**. The patient wears a HMD with head-tracking for immersion in the virtual environment from a first-person perspective of the avatar representing him. Wireless body tracking allows control over the avatar’s movements. A haptic device with force-feedback is used for tactile interaction with the environment and/or remote persons. Finally, several sensors and electrodes are attached to the patient to monitor his physiological and emotional state.

A medical scenario for remote rehabilitation has been implemented. The set-up has been conceived and designed for treating patients with motor deficits (e.g., stroke or Parkinson disease) or with neuropathic pain in upper limbs (e.g., complex regional pain syndrome, carpal tunnel syndrome). The objective is first to carry out an evaluation (and later a follow-up) of the neurological state of the patient, and next to guide him/her through the realization of rehabilitation exercises. It is also important to provide the patient the feeling of closeness to the medical team, contact (including physical) and support, even when the patient is at home. This is achieved by mediating the patient’s experience through immersive VR system (Figure 1). Once the patient is wearing the VR equipment, they are immersed in a hospital room where the medical personnel is actively represented. At the same time, the patient is “captured” from home and “beamed” into the hospital facilities, where his/her physical representation physically interacts with the hospital personnel. The specifications and technical details of this set-up for remote neurorehabilitation are described next. For a better understanding, the description of the set-up is divided into three subsections according to the two remote places (patient’s home and hospital), and a common platform for data exchange.

All the elements of the implemented set-up are shown in the Video S1 in Supplementary Material.

### The patient side

The patient connects to the application from home. After donning the VR equipment and physiological monitoring, the patient starts the session, becoming immersed into a virtual or mixed-reality representation of the doctor’s office. The embodiment of the patient into the virtual character is induced by means of the multisensory correlations and the body is seen by the patients from their first-person point of view, i.e., when they look down toward their body they will see a virtual body instead of their real one. The patient sees, hears, and touches as if he/she were at the doctor’s office. The exploration and evaluation of the patient takes place within this framework. We describe next the technical developments and equipment used at patient’s home that allow the immersive experience.

#### Virtual reality system

The VR system that the patient uses features a head-tracked stereoscopic HMD. The HMD is an NVIS SX111 with a resolution of 1280 × 1024 pixels per eye and a total field of view of 111° × 64°, displayed at 60 Hz. In order to have immersive VR it is necessary that when the participant moves the head, the graphics are updated for the new head position. In this way, once in the virtual environment, the participant can look around the environment as one can do in the real world. For this is necessary to have the position of the head-tracked. The head-tracking is obtained with a 6-DOF Intersense IS-900 device so that the movement of the patient results in the stream of video images updating in real time slaved to the head-gaze direction of the patient. As a consequence, the patient also would see the virtual body that represents him- or herself from a first-person perspective and substituting the place of his or her own body. Hence, when looking down toward their own body participants see a virtual body co-located with their own, and when they move, their virtual body will move accordingly (see Motion Capture of the Patient). This requires that both the head and the body are tracked in real time.

The patient sits in front of a computer to which the HMD and tracking devices are connected. The computer has an NVIDIA GeForce 480 GTX graphics card and uses NVDIA PhysX engine for virtual collision detection. The system was programmed using the XVR system (Tecchia et al., 2010) and the virtual body using the HALCA library (Gillies and Spanlang, 2010). The Arena software (Optitrack, NaturalPoint Inc.) is used for arms and upper body tracking to control the movements of the avatar. A gender-matched RocketBox (Rockebox Studios GmbH) avatar with 123 bones and articulated joints is used to represent the patient within the virtual environment.

#### Motion capture of the patient

The motion capture system is used to sample the body movements of the patient (in particular hands/arms). Hence, patients see their movements reflected in the co-located avatar representing them from a first-person perspective. This enables visuo-motor synchrony between their own movements and the movements that they see on the avatar (Gonzalez-Franco et al., 2010; Sanchez-Vives et al., 2010). Additionally, synchronous visuo-tactile stimulation may be applied to enhance the feeling of virtual body ownership over the virtual character. We define visuo-tactile stimulation as the tactile stimulation received at the real body of the participant while a synchronous virtual stimulus acts on the virtual body. Furthermore, allowing the patient to move within the virtual environment facilitates active tactile interaction with both virtual and remote persons and objects (see Haptic Interaction). The motion capture of the patients is critical not only for their embodiment within the virtual body representation but also for their representation at the hospital facilities, where they are represented by a virtual human displayed on a screen.

Two systems for body tracking are used:

a) Marker-based body tracking with Optitrack infrared cameras (NaturalPoint Inc.).
b) Markerless body tracking with Kinect (Microsoft Corporation).

Currently both systems have been integrated with our system, although marker-based tracking is preferred due to its higher resolution and reliability.

Optionally, an additional data glove can be used for tracking finger movements. The glove records flexion strength of fingers and uses this information to bend the avatar’s fingers individually, allowing opening/closing the virtual hand, grasping, or pointing to objects in the virtual environment.

Apart from being useful to induce virtual body ownership, all body tracking systems are of great value for the medical team to evaluate the evolution of the motor capabilities of the patients. Body tracking systems have the additional advantage over visual inspection that they can provide the position in space at any instant (trajectory tracking), therefore allowing an immediate quantification of different movement parameters such as amplitude or speed of movements, and their evolution from session to session.

#### Physiological measures

The set-up includes continuous recording and monitoring of the patient’s physiology, including electrocardiogram, galvanic skin response (GSR), and electromyographic activity of the affected limb. GSR is useful during exploration to reveal movements that may induce pain or discomfort. Additionally, the grip force in both hands is used for detecting force asymmetries and for the follow-up of the patient’s evolution. For that purpose, we have developed a device for measuring hand force built on pressure sensors, and have created a corresponding virtual model. All these data are recorded and streamed in real time to the remote doctor’s office (see The Data Exchange Platform). We have developed a stand-alone application for data monitoring and saving using Matlab (Mathworks Inc.). The physiological data are displayed at doctor’s PC screen (Figure 5).

More specialized measures could be integrated in the future, including nerve conduction velocity and pain threshold, among others.

#### Haptic interaction

A fundamental requirement for remote neurorehabilitation is to enable physical interaction between the medical staff and the patient. In particular we have used bidirectional haptic interaction including force-feedback. The novelty of the presented approach is not the interaction with the computer or virtual environment, but to enable person-to-person interaction between patient and doctor. Furthermore, this bidirectional haptic interaction enriches the sensory experience, amplifying the scope of tactile feedback and contributing to the illusory body feelings. Next we describe the haptic device for the physical task serving as a rehabilitation exercise, where biomechanical and neuromuscular interactions with the virtual environment and remote persons are enabled.

##### The GRAB device

The GRAB device is a mechanical arm with three actuated degrees of freedom, which can apply up to 20 N peak force (Figure 1). Both the device and the corresponding controller have been developed at the Scuola Superiore Sant’Anna in Pisa (Bergamasco et al., 2006). The haptic interface provides smooth, low-resistance movement in a large workspace (60 cm × 40 cm × 30 cm). The optimized kinematics and actuation systems supply high backdrivability and reduce the perceived inertia, these being fundamental properties for realistic teleoperation and force-feedback.

The device and the control software offer three configuration modes: single mode, teleoperation mode, and mixed mode. In the single mode, the device works as a joystick and the patient interacts with the virtual environment. The teleoperation mode allows bidirectional person-to-person interaction directly, reciprocally transmitting the forces applied locally to a second, remote unit. Two identical devices are therefore needed, in our case, one for the patient and one for the doctor. The most complete mode, however, is the mixed mode (a combination of the single and teleoperation modes), where both patient and doctor work together and that is especially useful for cooperative tasks, e.g., for lifting a virtual object. Therefore, when the mixed mode is enabled, the movement of the haptic device by the patient and the eventual collisions with virtual objects generate forces that are transmitted to the remote place, where the doctor feels these forces being applied remotely at the local haptic unit; and vice versa, the doctor may for instance explore the patient’s arm mobility remotely by moving his own haptic device, allowing the detection of resistances to movements. The bilateral teleoperation has been implemented according to a classical Position–Position (PP) scheme. Basically, the force-feedback for both the patient and the doctor are generated by a control based on the position error between the end effectors of the remote devices. Since the rehabilitation task is focused on the patient, the force-feedback generated by the collision between the ring and the wire is directly provided only to the patient. However, the doctor can still perceive the physical interaction with the virtual objects, since the position of his haptic device is coupled to the position of the patient’s interface.

**Figure 2:**
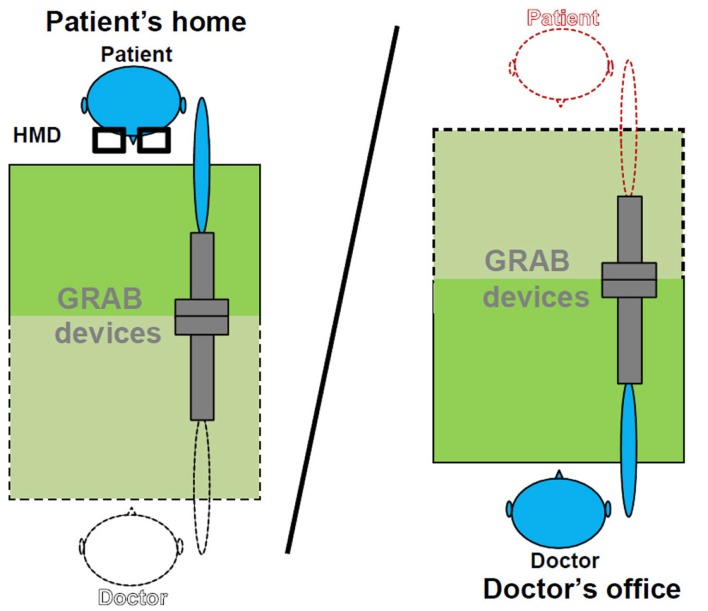
**Haptic interaction set-up in the mixed mode**. Objects displayed with black dot lines represent virtual objects seen through the HMD by the patient; objects drawn with continuous lines represent local objects at the corresponding side.

**Figure 3:**
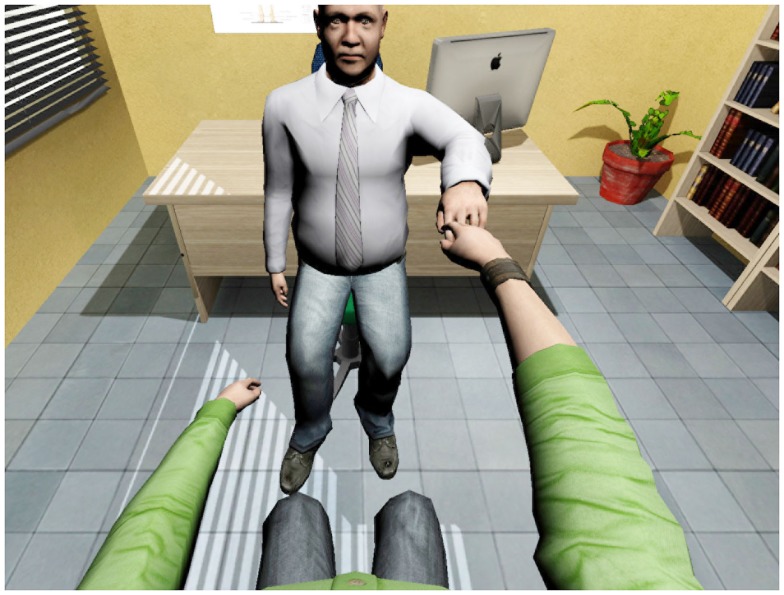
**Person-to-person haptic interaction with force-feedback**. The remote doctor explores patient’s arm mobility. The patient sees a virtual representation of the real doctor, which gives the hand to him.

Figure 2 shows the schema of the implemented set-up for haptic interaction. The picture on the left represents the described scenario at patient’s home. The patient wears the HMD and grabs the local haptic device with the fingertips or the palm using a handle. In the case the patient sees a virtual representation of the doctor, the doctor may hold either a virtual object or virtually take the hand of the patient’s virtual character (Figure 3).

##### The ring task

The ring task presents the implementation of a possible haptic task that could be used for evaluation of the patient’s performance or as an exercise for rehabilitation. It integrates a number of elements (virtual objects, force-feedback, tracking of trajectories, force monitoring) that could be combined in the design of different *ad hoc* tasks. Therefore, it should be taken as a model task for illustration.

**Figure 4:**
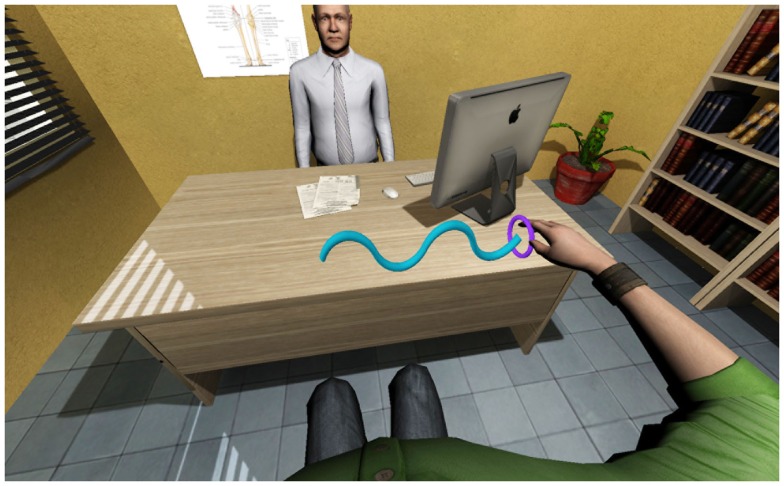
**The ring task**. A virtual ring is passed along a virtual wire without touching it. Whenever the wire is touched force-feedback is enabled. Camera view is from patient’s point of view (first-person perspective).

To enable physical interaction between the patient, the virtual environment and the doctor, a ring task has been implemented in the virtual environment as a rehabilitation exercise. The task consists of a virtual ring being passed along a virtual wire, with the aim of avoiding contact between the ring and the wire (Figure 4). The ring position is controlled with the haptic arm device at the patient’s side. Whenever the ring touches the virtual wire, the force-feedback is enabled so that the patient feels the collision and cannot go through the object. The physical simulation calculates the interaction forces between the virtual ring and the wire, while the position of the ring and the effector of the haptic device of the patient are linked by a virtual coupling with tuneable stiffness and damping.

The dynamical properties of the virtual ring, such as the mass and the friction with the wire, and the parameters of the PP bilateral teleoperation, such as the stiffness, can be set according to the capabilities of the patient.

### The hospital facilities

At the hospital facilities, the doctor monitors the session and patient’s physiological data, feels the presence of the patient and interacts with him or her.

#### Capture of the doctor’s office

Two options are considered here. First, a virtual model of the doctor’s office can be created based on captured images. The model is rendered in the HMD that the patient wears at home. The advantage is that this approach considerably reduces the computational load in data transmission to the patient’s side. The second option is to use the 3D video capturing for displaying the doctor’s office in the HMD in real time. This option allows the patient to follow those changes occurring at the doctor’s office in real time but at the expense of higher transmission bandwidths requirements. Currently both systems have been integrated with our system (Steptoe et al., 2012).

For tracking the hospital personnel, the same markerless tracking system based on Kinect implemented for the patient is used. In those cases where higher movement resolution or reliability may be required, the Optitrack system can be used in combination with inverse kinematics, so that only very few markers would be attached to the body (Badler et al., 1993).

**Figure 5:**
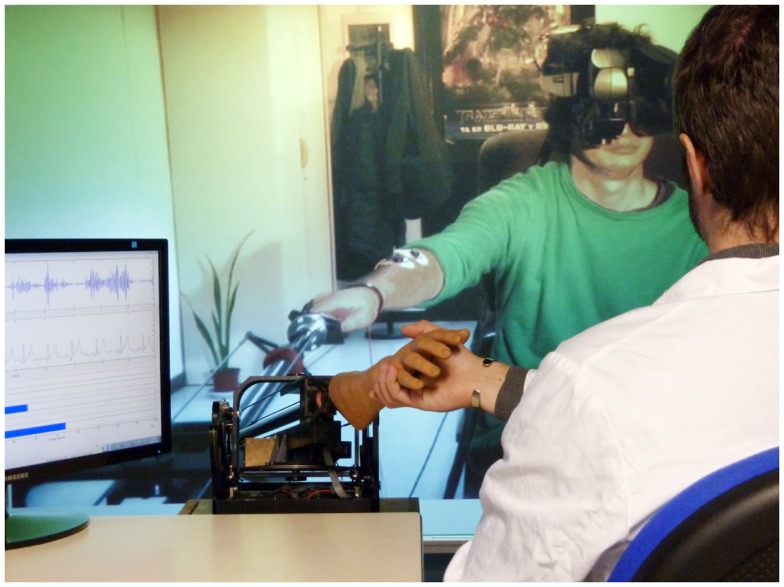
**The doctor’s office**. At the current set-up, the patient is displayed in a life-sized screen. A haptic device with force-feedback is used for tactile interaction with the patient. A PC screen is used for monitoring patient’s physiological data.

A GRAB device, like the one available at the patient’s home, is used in mixed teleoperation mode for haptic interaction with the patient. Besides the ring task, it may be useful to assess patient’s arm mobility or force, as the interconnected devices transfer the forces to each other in all three directions. The device is placed in a position that corresponds as far as possible with that at the patient’s home (Figure 5).

#### Representation of the patient

From a practical viewpoint, as a basic solution for the representation of the patient at the doctor’s office, life-sized 3D video is displayed on a 3D display (Figure 5). This solution requires the doctor to wear minimal equipment, at most some tracking device and/or 3D glasses for stereoscopic vision, allowing other doctors or hospital staff to join the session at any time easily. Flexibility is a key word in the proposed system. Therefore, an alternative solution, consisting in showing a virtual representation of the patient, is also contemplated (Steptoe et al., 2012).

### The data exchange platform

Data exchange between the patient’s side and the hospital is another critical aspect in the system. Different kinds of data need to be captured, computed, and transmitted from the origin (where they are originated) to the remote place (where they are displayed or reproduced) in real time. This problem is well-studied in the simulation and computer games domains (Steed and Oliveira, 2009). The technology is transferable to the telerehabilitation domain. Therefore, given the disparity of tracking and data acquisition systems available in the market, the safest way to assure that avatar data are displayed at both sides consistently is to compute all transformations (bones positions and orientations) locally and publish these data in a neutral, avatar-centered coordinate frame. Therefore, we have developed a software platform to centralize the management of data, including data transmission and reading to/from the connected clients, in such a way that the sent/read avatar data are exact clones. All sensory data streams (avatars, virtual scene, physiological data, speech, haptic information, etc.) travel from one physical place to another in parallel. The software platform is unique amongst its peers as it is designed to support a wide range of heterogeneous platforms, whilst retaining the ability to record and analyze interactions between participants (Steptoe et al., 2012).

#### Data security

The security of the physiological data processing and streaming is fundamental. There already exist several specific protocols to protect data in clinical as well as computer games networking environments. In particular, our platform is based on RAKNET (Jenkins Software LLC), a cross-platform, open-source C++ networking engine for game programming. RAKNET offers secure connectivity among other security measures, guarantying data privacy. An alternative, based on experience in the simulator or games industries, is to run the session over a virtual private network that can provide security for all services that are running between the both sides.

## Discussion

This is the first time, to our knowledge, that a telerehabilitation system based on immersive VR includes the internalization (and ownership) of whole virtual bodies. In particular, most current virtual rehabilitation systems do not integrate the full body into the virtual or mixed-reality environment (Mario et al., 2004; Tang et al., 2005) or present isolated virtual representations of the tracked hands only, interacting with the VE (Subramanian et al., 2007; August et al., 2011). In previous studies, when whole virtual body representations are considered, then they are in a collaborative non-immersive set-up (Kurillo et al., 2011). Therefore, having patients reacting to virtual and remote events as if they are real and happening locally are likely to lead to powerful illusions of place and plausibility of the situation (Slater, 2009), such that the participants are not mere spectators but become active actors (mentally and physically present) within the virtual environment. Consequently, these illusions should facilitate the natural interaction between the patient, the hospital personnel and, eventually, other patients, reducing concerns about both reduced social contact and reduced face-to-face contact with the therapist (Cranen et al., 2011). On the other hand, perceiving ownership of a virtual body opens the door for strategies of rehabilitation that are not possible when acting on the real body (Llobera et al., submitted).

In terms of haptic interaction, our novel approach stresses the direct tactile and force-feedback experience between patient and doctor, which enables a new range of functionalities, such as the remote exploration of limb mobility. Although some existing systems include unilateral (Popescu et al., 2000; Jadhav and Krovi, 2004) or bilateral (meaning cooperative; Carignan and Olsson, 2004) rehabilitation exercises, they always refer to direct interactions between the participant and the virtual environments only.

Regarding the representation of the patient at the hospital facilities, and as robotic technology becomes available, the patient may be embodied in a life-sized teleoperated agent through which the patient would be able to see, hear, feel the hospital environment, and it would also reproduce the patient’s movements at the hospital. The representation of the patient by a robot at the hospital will change the concept of remote tactile interaction as we know it today. At the hospital, sensors attached to different body parts of the robot would capture and transmit, when touched by the doctor, the generated tactile information to the patient’s body. There, the patient, attached with small actuators in the same body part as the robot (Kapur et al., 2010), will “feel” the contact, even with force-feedback if the patient wears the appropriate equipment (e.g., exoskeleton). Further, using a human-shaped robot as the physical representation of the patient, rather than a life-sized virtual avatar or a mechanical haptic device (representing patient’s arm), enhances the physical presence of the patient and, consequently, facilitates the natural interaction between the doctor and the patient. Indeed, robotic agents for remote embodiment are emerging. Recently, we have shown this concept using the same technology during an interview and demonstration at the BBC News (Laurence, 2012). Another good example is in Tachi et al. (2011).

Our motivation to develop such a technologically advanced system for neurorehabilitation is twofold. There is a demand from the medical community for improving rehabilitation tools (Levin, 2011), a need that will grow substantially over the coming decades as the population ages in the developed world. There is also a technological push from the commercialization and commoditization of technologies that were once restricted to laboratory settings (Huber et al., 2010; Lange et al., 2011). From a medical point of view there is a perpetual necessity for continuously improving the systems for rehabilitation as technology provides new tools for expanding the effectiveness and target patient population, and neuroscience offers new insights in the plastic capabilities of the brain (Levin, 2011). From a technical point of view, the development and rapid standardization of technological advances offers an increasing number of devices and tools (maybe not primarily thought for medical use) that improve efficiency and possibilities of current instrumental used in the clinic (Huber et al., 2010; Lange et al., 2010).

Here we have described a frame for remote neurorehabilitation and implemented a prototype in the laboratory, but it could be reproduced in the future in more affordable systems (e.g., home-based system). The accelerated emergence of consumer devices for entertainment and computer gaming, such as HMDs, tracking systems, 3D screens, or mobile robots, among others, may soon allow the integration of such systems anywhere, including the patient’s home (Lange et al., 2012). Although in most cases these consumer devices do not offer the precision and reliability of those specifically designed for professional and research purposes yet (Lange et al., 2009, 2011), they are of great utility since they allow rapid testing of the desired concept or effect during preliminary stages, before investing large amounts of money in projects whose long-term profitability and cost-effectiveness has yet to be proved. We further expect markerless body tracking systems to significantly improve in the near future, eventually widely replacing marker-based systems thanks to their non-invasive and inexpensive character.

Bringing today’s computer science, VR, robotics, and neuroscience together does not assure the success of the venture *per se* (Kenyon et al., 2004). In order to assure a long-term success in the standardization and integration of emerging technologies in society, they need to go hand-by-hand with a wide, global theoretical approach that considers all factors involved, including economical and ethical. For example, the viability of non-profitable solutions, economically speaking, becomes problematic, independent of the necessity or contribution to the well-being of humanity. Saving emissions and costs (Rimmer et al., 2008) and improving efficiency and usability (Cranen et al., 2011) have become as essential as medical factors. Truly effective telemedicine and telepresence technologies hope to reduce the need for travel (CO_2_ release, time, and energy savings) by augmenting effective communication dramatically in comparison to current solutions (Vespa et al., 2007).

In such a technology-based approach, factors such as the cognitive-emotional and social context should also be taken into consideration for successful participant-acceptance (Buck, 2009). In particular, in-home patients often suffer from solitude and isolation (Cranen et al., 2011), additionally to their physical problems, what may aggravate their personal situation. Therefore, the possible social role of the proposed set-up for patients with motion difficulties needs to be evaluated. A waiting room scenario for the patient to wait in prior to the doctor’s appointment, similar to a real-life scenario, is envisaged. The patient, wearing the VR equipment, is virtually beamed into the doctor’s waiting room and represented by his/her own controlled avatar. There he/she can move around and interact with other patients also waiting there, while waiting for his doctor’s appointment. The assessment of the effectiveness of all these scenarios through participant studies is envisaged.

Beyond the technology itself, there are open questions concerning the display of remote places and persons that may affect participant’s experience: should the patient be virtually represented or physically embodied in a robot? Should the real physical doctor’s office be displayed or may it be replaced with a neutral or emotionally relevant (e.g., peaceful landscape)? In order to answer these questions participant studies should follow. Here the versatility of VR facilitates such comparisons for evaluating participant’s preferences in each case.

## Summary

In this paper, we have presented an innovative set-up for remote interaction and remote rehabilitation that includes the body projection into virtual bodies in a fully immersive environment and physical embodiment at the remote place. This unique system for telerehabilitation is the result of the integration of state of the art technologies developed at different institutions in the fields of VR, haptics, computer science, biomedical research, and neuroscience. This approach systematically differs from non-immersive telerehabilitation systems and should represent a step forward in the field.

## Conflict of Interest Statement

The authors declare that the research was conducted in the absence of any commercial or financial relationships that could be construed as a potential conflict of interest.

## Supplementary Material

The Video S1 for this article can be found online at http://www.frontiersin.org/Teleneurology/10.3389/fneur.2012.00110/abstract
